# Supplementary material for: Infection and Risk Perception of SARS-CoV-2 among Airport Workers: A Mixed Methods Study
Source: Int J Environ Res Public Health. 2020 Dec 3;17(23):9002. doi: 10.3390/ijerph17239002 (PMC7730724; doi:10.3390/ijerph17239002)
Supplement: Supplementary file 1 [file ijerph-17-09002-s001.zip › ijerph-977920-Supplementary/Supplementary material.pdf]

Supplementary figure 1. Hazard function for SARS-CoV-2 infection among aiport workers

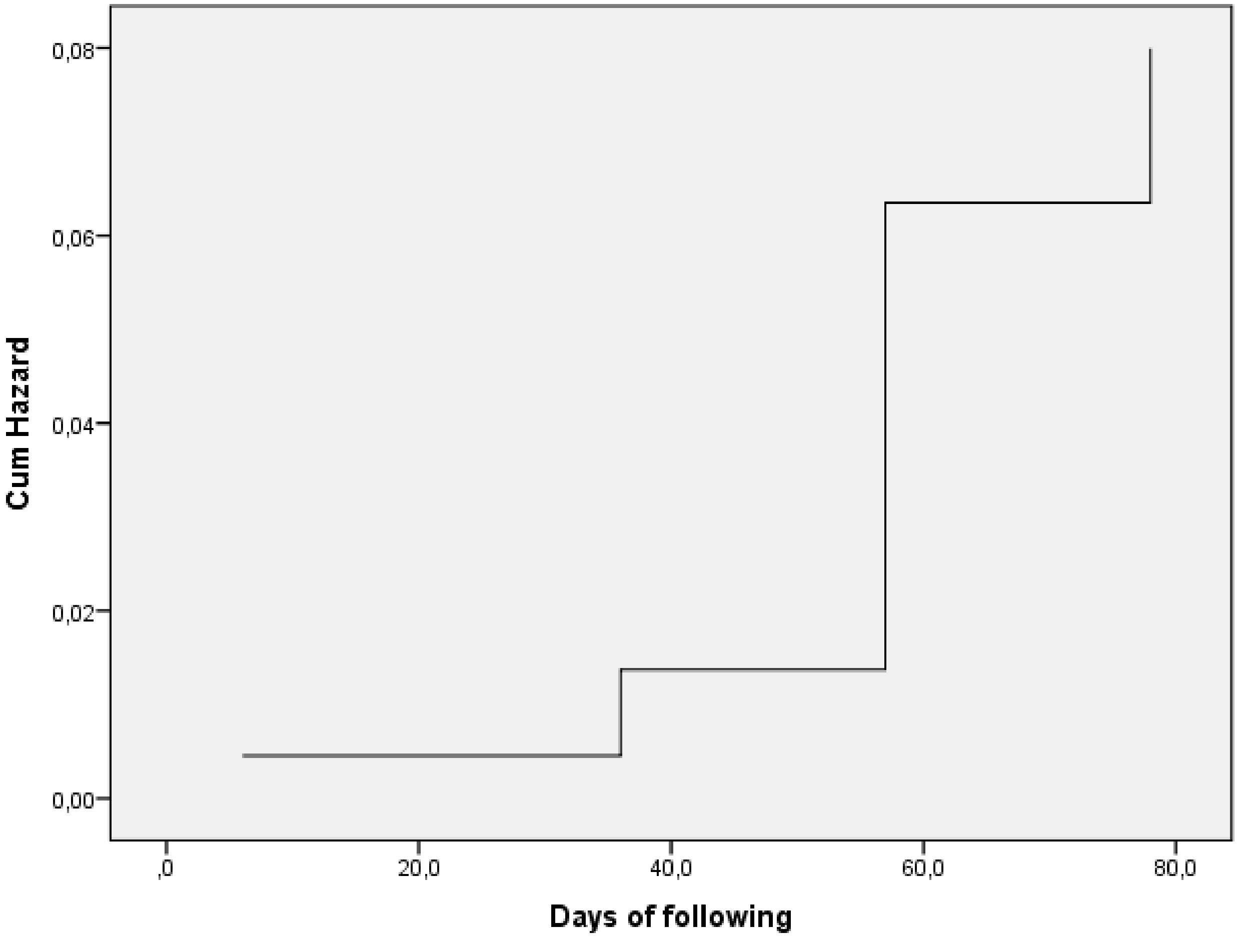

Supplementary table1. Sample size distrtibution by area

Area	n	Proportion	n
**Cargo**	140	28%	58
**Security**	100	20%	40
**Administrative**	60	12%	24
**Airline ground staff**	70	14%	29
**General services**	60	12%	24
**Technological services**	40	8%	16
**Health and emergencies**	20	4%	8
**Others**	15	3%	6
**Total**	505	100%	205

Supplementary Table 2. Sociodemographic characteristics of close contacts.

Age range (Years)	Female n (%)		Male n (%)	
**0-5**	5	(13.5%)	4	(10.8%)
**6 – 17**	3	(8.1%)	2	(5.4%)
**18 - 50**	9	(24.3%)	6	(16.2%)
**51 - 60**	2	(5.4%)	4	(10.8%)
**> 60**	1	(2.7%)	1	(2.7%)
**Total**	20	(54.1%)	17	(45.9%)

STROBE Statement—checklist of items that should be included in reports of observational studies

	Item No.	Recommendation	Page No.	Relevant text from manuscript
**Title and abstract**	1	(*a*) Indicate the study's design with a commonly used term in the title or the abstract	1	
		(*b*) Provide in the abstract an informative and balanced summary of what was done and what was found	1	
**Introduction**				
Background/rationale	2	Explain the scientific background and rationale for the investigation being reported	1	
Objectives	3	State specific objectives, including any prespecified hypotheses	2	
**Methods**				
Study design	4	Present key elements of study design early in the paper	2	
Setting	5	Describe the setting, locations, and relevant dates, including periods of recruitment, exposure, follow-up, and data collection	2	
Participants	6	(*a*) *Cohort study*—Give the eligibility criteria, and the sources and methods of selection of participants. Describe methods of follow-up<br>*Case-control study*—Give the eligibility criteria, and the sources and methods of case ascertainment and control selection. Give the rationale for the choice of cases and controls<br>*Cross-sectional study*—Give the eligibility criteria, and the sources and methods of selection of participants	2	
		(*b*) *Cohort study*—For matched studies, give matching criteria and number of exposed and unexposed<br>*Case-control study*—For matched studies, give matching criteria and the number of controls per case		
Variables	7	Clearly define all outcomes, exposures, predictors, potential confounders, and effect modifiers. Give diagnostic criteria, if applicable	3	
Data sources/ measurement	8*	For each variable of interest, give sources of data and details of methods of assessment (measurement). Describe comparability of assessment methods if there is more than one group	*3-4*	
Bias	9	Describe any efforts to address potential sources of bias	3-4	
Study size	10	Explain how the study size was arrived at		

Continued on next page

Quantitative variables	11	Explain how quantitative variables were handled in the analyses. If applicable, describe which groupings were chosen and why	4-5
Statistical methods	12	(*a*) Describe all statistical methods, including those used to control for confounding	5
		(*b*) Describe any methods used to examine subgroups and interactions	5
		(*c*) Explain how missing data were addressed	5-6
		(*d*) *Cohort study*—If applicable, explain how loss to follow-up was addressed<br>*Case-control study*—If applicable, explain how matching of cases and controls was addressed<br>*Cross-sectional study*—If applicable, describe analytical methods taking account of sampling strategy	
		(*e*) Describe any sensitivity analyses	
**Results**			
Participants	13*	(a) Report numbers of individuals at each stage of study—eg numbers potentially eligible, examined for eligibility, confirmed eligible, included in the study, completing follow-up, and analysed	7-8
		(b) Give reasons for non-participation at each stage	N.A.
		(c) Consider use of a flow diagram	Figure 1
Descriptive data	14*	(a) Give characteristics of study participants (eg demographic, clinical, social) and information on exposures and potential confounders	8
		(b) Indicate number of participants with missing data for each variable of interest	
		(c) *Cohort study*—Summarise follow-up time (eg, average and total amount)	8
Outcome data	15*	*Cohort study*—Report numbers of outcome events or summary measures over time	*8-9*
		Case-control study—Report numbers in each exposure category, or summary measures of exposure	
		Cross-sectional study—Report numbers of outcome events or summary measures	
Main results	16	(*a*) Give unadjusted estimates and, if applicable, confounder-adjusted estimates and their precision (eg, 95% confidence interval). Make clear which confounders were adjusted for and why they were included	8-10
		(*b*) Report category boundaries when continuous variables were categorized	
		(*c*) If relevant, consider translating estimates of relative risk into absolute risk for a meaningful time period	

Continued on next page

Other analyses	17	Report other analyses done—eg analyses of subgroups and interactions, and sensitivity analyses	10
**Discussion**			
Key results	18	Summarise key results with reference to study objectives	15
Limitations	19	Discuss limitations of the study, taking into account sources of potential bias or imprecision. Discuss both direction and magnitude of any potential bias	16
Interpretation	20	Give a cautious overall interpretation of results considering objectives, limitations, multiplicity of analyses, results from similar studies, and other relevant evidence	16
Generalisability	21	Discuss the generalisability (external validity) of the study results	
**Other information**			
Funding	22	Give the source of funding and the role of the funders for the present study and, if applicable, for the original study on which the present article is based	17

*Give information separately for cases and controls in case-control studies and, if applicable, for exposed and unexposed groups in cohort and cross-sectional studies.

Note: An Explanation and Elaboration article discusses each checklist item and gives methodological background and published examples of transparent reporting. The STROBE checklist is best used in conjunction with this article (freely available on the Web sites of PLoS Medicine at http://www.plosmedicine.org/, Annals of Internal Medicine at http://www.annals.org/, and Epidemiology at http://www.epidem.com/). Information on the STROBE Initiative is available at www.strobe-statement.org.

Manuscript title:	Yes	No	Page number (if not applicable, indicate NA)
**Title**			
1. Does the title directly indicate or sufficiently allude to the methodological contribution of the article?	X	☐	1
**Abstract**			
2. Does the abstract include an explicit statement about a methodological challenge or issue in the field that will be addressed in the article?	X	☐	1
3. Does the abstract indicate the methodological/theoretical contribution of the article to the field of mixed methods research?	X	☐	1
**Main text of the article**			
4. Does the article have a clear writing style with sufficient headers and sub-headers such that the reader can readily follow the flow and argumentation?	X	☐	2-5
5. Does the text in the background reiterate and expand upon the methodological challenge or issue as identified in the abstract?	X	☐	2-5
6. Does the background contain a rigorous review and citations of relevant and recent mixed methods literature to support examining the methodological aim?	X	☐	2-5
7. Does the background include an explicit methodological aim?	X	☐	3-9
8. Does the background contain an explication of the article's structure and methodological points that will be addressed?	X	☐	3
9. In the body of article, are each of the methodological points identified in #8 addressed persuasively in the order specified?	X	☐	3-5
10. Does the article include a strategy to convey the overall complexity of the topic or study phenomenon such as a figure or illustration?	X	☐	1-14
11. In the discussion, are the explicit points made in #8 synthesized together to logically support the overarching methodological aim?	X	☐	3
12. Does the discussion section include a specific subsection "Contribution to the Field of Mixed Methods Research" that reviews the points made and extant literature to articulate the articles novel contribution(s) to mixed methods?	X	☐	14-16
13. Does the article have a discussion of the methodological limitations?	X	☐	16
14. Does the discussion section include recommendations for future mixed methods inquiry based on the paper's unique contribution or limitations?	X	☐	16
15. Have the references been cited according to the current American Psychological Association style?	X	☐	16
**Additional elements for empirical methodological articles only**			
16. Does the background of the article include explicit statements of both the methodological aim and purpose of the empirical study separately?	X	☐	3-9
17. Does the description of the methods include sufficient detail about the procedures used and present these in a logical order?	X	☐	3-9
18. Does the submission include a procedural diagram of the data collection and analysis procedures as a figure?	X	☐	3-9 3
19. Does the submission include a table, matrix or visual structure, e.g., joint display, to illustrate integration and interpretation of the qualitative and quantitative findings?	X	☐	3
20. Does the discussion articulate how the use of a mixed methods approach advanced a greater understanding of the substantive topic compared to using a monomethod approach?	X	☐	16-17

Note. Adapted from Fetters and Freshwater (2015a) and Fetters and Molina-Azorin (2019).
