# Supplementary material for: Infection and Risk Perception of SARS-CoV-2 among Airport Workers: A Mixed Methods Study
Source: Int J Environ Res Public Health. 2020 Dec 3;17(23):9002. doi: 10.3390/ijerph17239002 (PMC7730724; doi:10.3390/ijerph17239002)
Supplement: Supplementary file 1 [file ijerph-17-09002-s001.zip › ijerph-977920-Supplementary/Supplementary Table 1 v 29.10.20.pdf]

Supplementary table1. Sample size distrtibution by area

Area	n	Proportion	n
**Cargo**	140	28%	58
**Security**	100	20%	40
**Administrative**	60	12%	24
**Airline ground staff**	70	14%	29
**General services**	60	12%	24
**Technological services**	40	8%	16
**Health and emergencies**	20	4%	8
**Others**	15	3%	6
**Total**	505	100%	205
