# Supplementary material for: Infection and Risk Perception of SARS-CoV-2 among Airport Workers: A Mixed Methods Study
Source: Int J Environ Res Public Health. 2020 Dec 3;17(23):9002. doi: 10.3390/ijerph17239002 (PMC7730724; doi:10.3390/ijerph17239002)
Supplement: Supplementary file 1 [file ijerph-17-09002-s001.zip › ijerph-977920-Supplementary/Supplementary Table 2v 29.10.20.pdf]

Supplementary Table 2. Sociodemographic characteristics of close contacts.

Age range (Years)	Female n (%)		Male n (%)	
**0-5**	5	(13.5%)	4	(10.8%)
**6 – 17**	3	(8.1%)	2	(5.4%)
**18 - 50**	9	(24.3%)	6	(16.2%)
**51 - 60**	2	(5.4%)	4	(10.8%)
**> 60**	1	(2.7%)	1	(2.7%)
**Total**	20	(54.1%)	17	(45.9%)
